# *Leptospira* species and serovars identified by MALDI-TOF mass spectrometry after database implementation

**DOI:** 10.1186/1756-0500-7-330

**Published:** 2014-06-02

**Authors:** Adriana Calderaro, Giovanna Piccolo, Chiara Gorrini, Sara Montecchini, Mirko Buttrini, Sabina Rossi, Maddalena Piergianni, Flora De Conto, Maria Cristina Arcangeletti, Carlo Chezzi, Maria Cristina Medici

**Affiliations:** 1 Unit of Microbiology and Virology, Department of Clinical and Experimental Medicine, University of Parma, Viale A. Gramsci, 14-43126 Parma, Italy

**Keywords:** *Leptospira* sp., MALDI-TOF MS, Identification, Database implementation

## Abstract

**Background:**

Leptospirosis, a spirochaetal zoonotic disease of worldwide distribution, endemic in Europe, has been recognized as an important emerging infectious disease, though yet it is mostly a neglected disease which imparts its greatest burden on impoverished populations from developing countries. Leptospirosis is caused by the infection with any of the more than 230 serovars of pathogenic *Leptospira* sp. In this study we aimed to implement the MALDI-TOF mass spectrometry (MS) database currently available in our laboratory with *Leptospira* reference pathogenic (*L. interrogans*, *L. borgpetersenii*, *L. kirschneri*, *L. noguchii*), intermediate (*L. fainei*) and saprophytic (*L. biflexa*) strains of our collection in order to evaluate its possible application to the diagnosis of leptospirosis and to the typing of strains. This was done with the goal of understanding whether this methodology could be used as a tool for the identification of *Leptospira* strains, not only at species level for diagnostic purposes, but also at serovar level for epidemiological purposes, overcoming the limits of serological and molecular conventional methods. Twenty *Leptospira* reference strains were analysed by MALDI-TOF MS. Statistical analysis of the protein spectra was performed by ClinProTools software.

**Results:**

The spectra obtained by the analysis of the reference strains tested were grouped into 6 main classes corresponding to the species analysed, highlighting species-specific protein profiles. Moreover, the statistical analysis of the spectra identified discriminatory peaks to recognize *Leptospira* strains also at serovar level extending previously published data.

**Conclusions:**

In conclusion, we confirmed that MALDI-TOF MS could be a powerful tool for research and diagnostic in the field of leptospirosis with broad applications ranging from the detection and identification of pathogenic leptospires for diagnostic purposes to the typing of pathogenic and non-pathogenic leptospires for epidemiological purposes in order to enrich our knowledge about the epidemiology of the infection in different areas and generate control strategies.

## Background

Leptospirosis, a spirochaetal zoonotic disease of worldwide distribution [1,2], endemic in Europe, has been recognized as an important emerging infectious disease [3], though yet it is mostly a neglected disease which imparts its greatest burden on impoverished populations from developing countries [2]. More than 1 million cases of severe leptospirosis occur worldwide annually [4] and the disease varies from a sub clinical infection to a severe illness with multi-organ involvement, leading to a fatal form in some cases. Leptospirosis is caused by the infection with any of the more than 230 serovars of pathogenic *Leptospira* sp. [4], clustered into 24 serogroups as a function of antigenic determinants and into 20 genomospecies, on the basis of genetic methods [3,5,6]. Evidence was demonstrated that serogrouping does not strictly equate with speciation, because some serovars within the same serogroup may be distributed among different species [5,6].

The identification of the causative *Leptospira* isolate at the species level allows to determine its pathogenic status, the probable source of infection and to distinguish sporadic cases from outbreaks [7]. The gold standard methodology for leptospirosis diagnosis, the microscopic agglutination test (M.A.T.), is based on the antibody response of the host, which can occur only in a period of 8–10 days after the onset of symptoms (seroconversion) [8], and it presents the inconvenience of being laborious and requires the maintenance of living cultures. Serotyping requires an indaginous technical procedure providing an ambiguous identification of *Leptospira* isolates and it is performed only in reference laboratories [7]. Moreover, several molecular assays till now proposed [5,9-13] cannot be routinely applied to the identification of *Leptospira* isolates since they do not allow for unambiguous identification of *Leptospira* isolates, needing for sequencing-based techniques [6,7,14-19].

Matrix-assisted laser desorption ionization-time-of-flight mass spectrometry (MALDI-TOF MS) emerged over the last years as a first line technique for the rapid bacterial identification [20,21]. Recently, in a study by our research group [22], MALDI-TOF MS was applied for the first time on *Brachyspira* strains of human and animal origins, overcoming the problems previously encountered in the identification of these spirochaetes when using biochemical and genetic-based methods. Furthermore, we also applied this technology to the identification of *Borrelia* sp. strains at species level [23].

In the field of leptospirosis, Djelouadji et al. [7] and Rettinger et al. [24] evaluated the application of MALDI-TOF MS for the identification of *Leptospira* sp. strains. In this study, performed in our Regional Reference Laboratory for Leptospirosis of the Italian Ministry of Health we analysed our own panel of *Leptospira* reference pathogenic (*L. interrogans*, *L. borgpetersenii*, *L. kirschneri*, *L. noguchii*), intermediate (*L. fainei*) and saprophytic (*L. biflexa*) strains circulating in Italy with the aim to supplement our MALDI-TOF database. In this way, the possible application of the MALDI-TOF MS to the diagnosis of leptospirosis and to the typing of strains was evaluated including both *Leptospira* strains already tested at species level by previous published papers [7,24] and *Leptospira* strains not investigated before. This was done with the intent to make a further contribution to understand whether this methodology could be used as a tool for the identification of *Leptospira* strains, not only at species level for diagnostic purposes, but also at serovar level for epidemiological purposes, overcoming the limits of serological and molecular conventional methods.

## Methods

### Leptospira strains

A total of 20 *Leptospira* reference strains, 18 provided for diagnostic purposes (these strains are also authorized to be utilized for research use) from the Istituto Superiore di Sanità, Rome, Italy, National Reference Laboratory for Leptospirosis (*L. interrogans*, *L. borgpetersenii*, *L. kirschneri* and *L. biflexa*) and from the Centre National des Leptospires, Institut Pasteur, Paris (*L. noguchii* and *L. fainei*), were included in this study (Table 1).

**Table 1:** **Twenty *Leptospira *** **reference serovars belonging to 6 species used for MALDI-TOF MS analysis**

**No.**	**Serovar**	**Serogroup**	**Strain**	**Genomospecies**	**Pathogenicity**
1	Autumnalis	Autumnalis	Akiyami A	*L. interrogans*	Pathogenic
2	Bataviae	Bataviae	Pavia 1	*L. interrogans*	Pathogenic
3	Bratislava	Australis	Riccio2	*L. interrogans*	Pathogenic
4	Canicola	Canicola	Alarik	*L. interrogans*	Pathogenic
5	Copenhageni	Icterohaemorrhagiae	Wijnberg	*L. interrogans*	Pathogenic
6	Hardjo	Sejroe	Hardjoprajitno	*L. interrogans*	Pathogenic
7	Hardjo	Sejroe	Farina C. 715	*L. interrogans*	Pathogenic
8	Hebdomadis	Hebdomadis	Hebdomadis H	*L. interrogans*	Pathogenic
9	Icterohaemorrhagiae	Icterohaemorrhagiae	Bianchi 1	*L. interrogans*	Pathogenic
10	Lora	Australis	Riccio 37	*L. interrogans*	Pathogenic
11	Pomona	Pomona	Mezzano I	*L. interrogans*	Pathogenic
12	Saxkoebing	Sejroe	Mus 24	*L. interrogans*	Pathogenic
13	Zanoni	Pyrogenes	Zanoni	*L. interrogans*	Pathogenic
14	Sejroe	Sejroe	Topo 1	*L. borgpetersenii*	Pathogenic
15	Mini	Mini	Sari	*L. borgpetersenii*	Pathogenic
16	Castellonis	Ballum	Castellon 3	*L. borgpetersenii*	Pathogenic
17	Grippotyphosa	Grippotyphosa	Moskva V	*L. kirschneri*	Pathogenic
18	Panama	Panama	CZ 214 K	*L. noguchii*	Pathogenic
19	Hurstbridge	Hurstbridge	BUT6	*L. fainei*	Intermediate
20	Patoc	Semaranga	Patoc 1	*L. biflexa*	Saprophytic

According to Brenner et al. [5].

These strains were cultured on Ellinghausen-McCullough Johnson and Harris (EMJH) medium (Difco^TM^ Leptospira Medium Base EMJH and Difco^TM^ Leptospira Enrichment EMJH BD, USA) at 30°C [8]. Two different lots of this medium (lots no. 1311945 and no. 2299369) were tested in order to ensure the reproducibility of protein profiles. Microscopic observation was performed to exclude the presence of microorganisms other than leptospires and to appreciate the *Leptospira* sp. growth in cultures that were periodically sub-cultured into fresh media. Bacteria used for MALDI-TOF MS measurements and for the creation of protein reference spectra were cultured for 7 days.

The strains belonging to the pathogenic species *L. interrogans*, *L. borgpetersenii* and *L. kirschneri* are currently used to perform the M.A.T. in our laboratory that is the Regional Reference Laboratory for Leptospirosis of the Italian Ministry of Health.

### MALDI-TOF MS analysis

After 7 days of growth, bacteria were counted by microscopy and an aliquot of 1 ml containing at least 1 × 10^6^ leptospires per ml was utilized for ethanol/formic acid extraction, as previously described [22]. The obtained extracts were spotted 40 times onto a “MSP-96 polished steel” target plate (Bruker Daltonics, Germany). Immediately after drying, all spots were overlaid with 1 μl of matrix, a saturated solution of α-cyano-4-hydroxycinnamic acid (HCCA) (Bruker Daltonics, Germany) dissolved in 50% CAN and 2.5% trifluoroacetic acid (TFA) (Sigma-Aldrich, Milan, Italy).

In each plate *Escherichia coli* (ATCC 8739) was used as a positive control and a non-inoculated matrix solution was used as a negative control. Each spot was measured in 40-shot steps for a total of 240 laser shots. MALDI-TOF MS measurements were performed by a Microflex LT mass spectrometer (Bruker Daltonics, Germany) provided with a 20 Hz nitrogen laser using FlexControl software (version 3.3.63, Bruker Daltonics, Germany). Spectra were obtained in the linear positive mode with an accelerating voltage of 20 kV and analysed within a mass range of 2,000-20,000 Da. Pre-processing and identification steps were performed using the manufacturer’s parameters. Before each measurement, the instrument was calibrated using the Bacterial Test Standard (BTS) provided by Bruker Daltonics. Preparation of the BTS and calibration were performed following the manufacturer’s instructions: calibration was successful when proteins of the mass spectra were in a range of ±300 parts per million (ppm).

Results of the pattern-matching process were expressed as identifying scores varying from 0 to 3.0; a score of ≥ 2.0 indicates a species level identification; a score of 1.7-2.0 indicates a genus level identification and a score of ≤ 1.7 is regarded as an unreliable identification.

### MALDI-TOF MS database and dendrogram

MALDI-TOF MS analysis of reference *Leptospira* strains representative of 6 species (*L. interrogans*, *L. kirschneri*, *L. borgpetersenii*, *L. biflexa*, *L. noguchii,* and *L. fainei*) was performed.

A total of 40 replicates for each strain were performed and the obtained spectra were analysed with Flex Analysis software (Version 3.3). For each of the strains belonging to these 6 species, a MSP (Main Spectrum Profile) spectrum was obtained using the MALDI-Biotyper software (Bruker Daltonics, Germany) and then loaded in the Bruker Daltonics database (version 3.1.2.0). Subsequently, a blind trial was performed by using the 20 *Leptospira* strains included in the database.

Spectra obtained in this study were matched with the commercial Bruker database previously added by us with spectra from *Brachyspira* sp. and *Borrelia* sp. [22,23].

Cluster analysis was performed based on comparison of strain specific main spectra (MSP dendrogram) created as previously described [22].

### ClinProTools statistical analysis

The same MALDI-TOF MS spectra previously selected for the database implementation were re-analysed with Flex Analysis software to perform the “smoothing” and the “baseline” for each spectrum. Subsequently, in order to compare the supplemented spectra and to identify specific discriminating peaks within the different analysed serovars, a statistical analysis was performed.

Then, the obtained spectra were loaded in an equal number in ClinProTools software version 2.2 (Bruker Daltonics) automatically recalibrated.

The analysis was performed within the range from 3,000 to 12,000 Da. To compare each strains the following specific algorithms of the software were used: Quick Classifier (QC) and Supervised Neural Network (SNN), that automatically give the highest value of “recognition capability (RC)” together with the highest value of “cross validation (CV)” with the highest number of peaks (from 1 to 25), and the Genetic Algorithm that gives a value of RC together with a value of CV based on the maximum number of peaks selected by the operator. The classifications obtained with the 3 models were compared and the spectra considered to be different by at least 2 of the 3 models were recognized as “outliers” and eliminated. Moreover, in order to standardize the number of spectra analysed for each strain of each tested species, after the removal of the “outliers”, 20 spectra were selected and then re-analysed by the same 3 statistical algorithms which displayed a list of discriminating peaks for the analysed spectra according to the selected algorithm. The algorithm with the highest score of RC and the highest value of CV, taking into account also the number of the peaks utilized to obtain the model, was chosen to analyze the discriminating peaks that were displayed in a report. The presence or the absence of each discriminating peak was evaluated by comparing each average spectra automatically created from the replicates of each strain with the total average spectrum created with all the replicates loaded in the software.

## Results

### MALDI-TOF MS database and dendrogram

*Leptospira* protein profiles obtained in this study were found to be original, matching none of the existing profile in the database (score of the best match < 1.3), when compared with the Bruker Daltonics database containing about 4,000 spectra corresponding to more than 2,000 bacterial species, including *Brachyspira* sp. and *Borrelia* sp. [22,23].

The spectra obtained by MALDI-TOF MS automatic acquisition were analysed by using Flex Analysis software. The replicates with an intensity <10^4^ arbitrary units as well as those with a profile highly different from the others were discarded.

**Figure 1:**
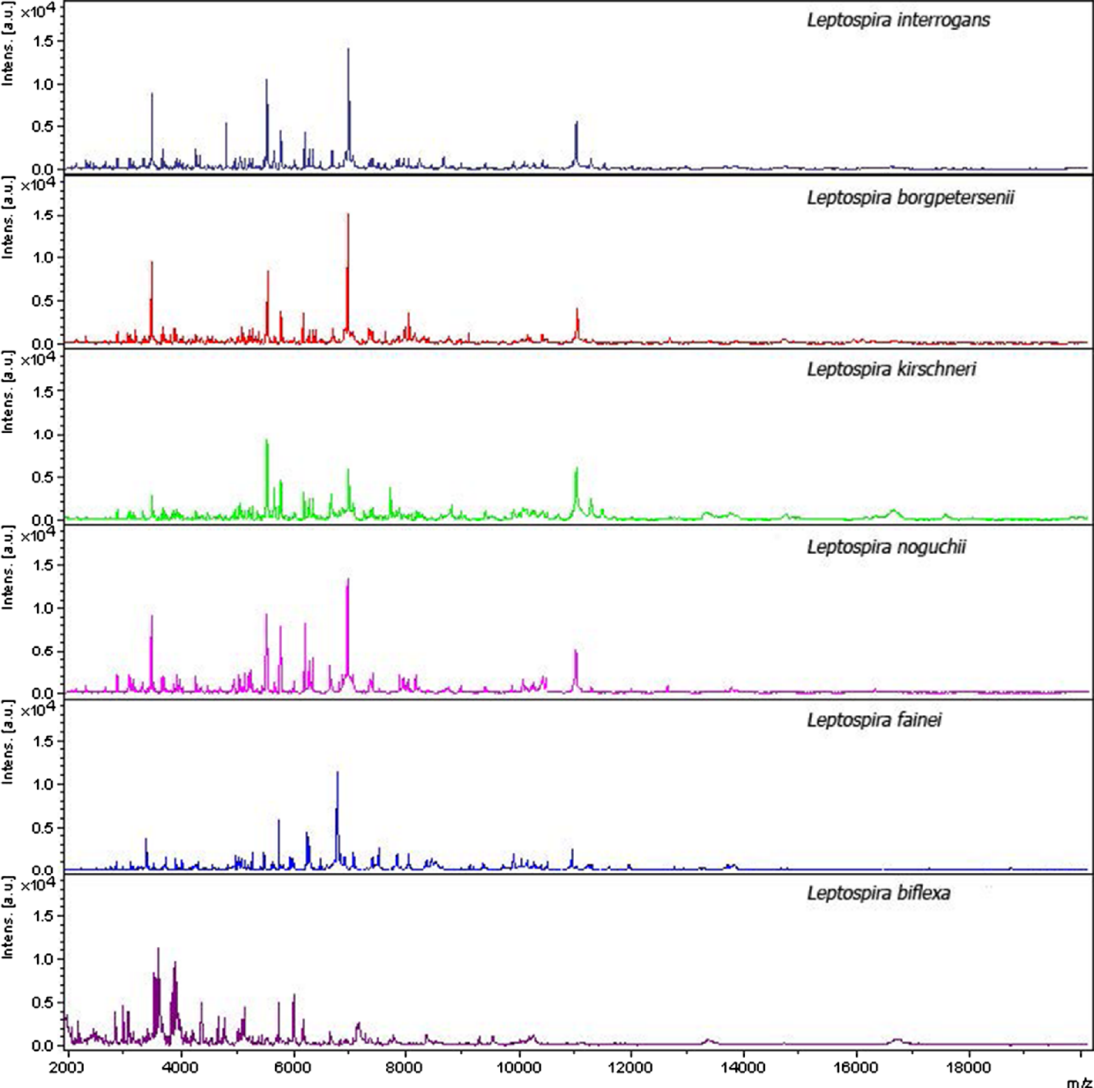
**Spectra obtained by analysing reference strains of the 6 *Leptospira *** **species by MALDI-TOF MS.**

The spectra obtained by the analysis of the 20 reference strains tested were grouped into 6 main classes corresponding to the 6 species *L. interrogans*, *L. borgpetersenii*, *L. kirschneri*, *L. biflexa*, *L. noguchii*, and *L. fainei* (Figure 1), highlighting species-specific protein profiles with unique peaks in each species analysed.

The replicates, previously selected by Flex Analysis software, were used to create a MSP spectrum for each reference strain using MALDI-Biotyper software. The obtained MSP spectra were supplemented in our Bruker Daltonics database in order to identify *Leptospira* strains at the species level. These profiles proved to be reproducible in a second independent experiment performed after the database supplementation and a correct identification at the species and the serovar level was found for all replicates of each strain (score of the best match > 2.3).

This reproducibility was observed with different lots of EMJH medium; moreover, each session was validated by external negative and positive controls: in all experiments the negative control spots yielded no peaks or faint profiles which were not identifiable by the system, and the positive control spots yielded *E. coli* identification with an identification score of 2–2.5.

**Figure 2:**
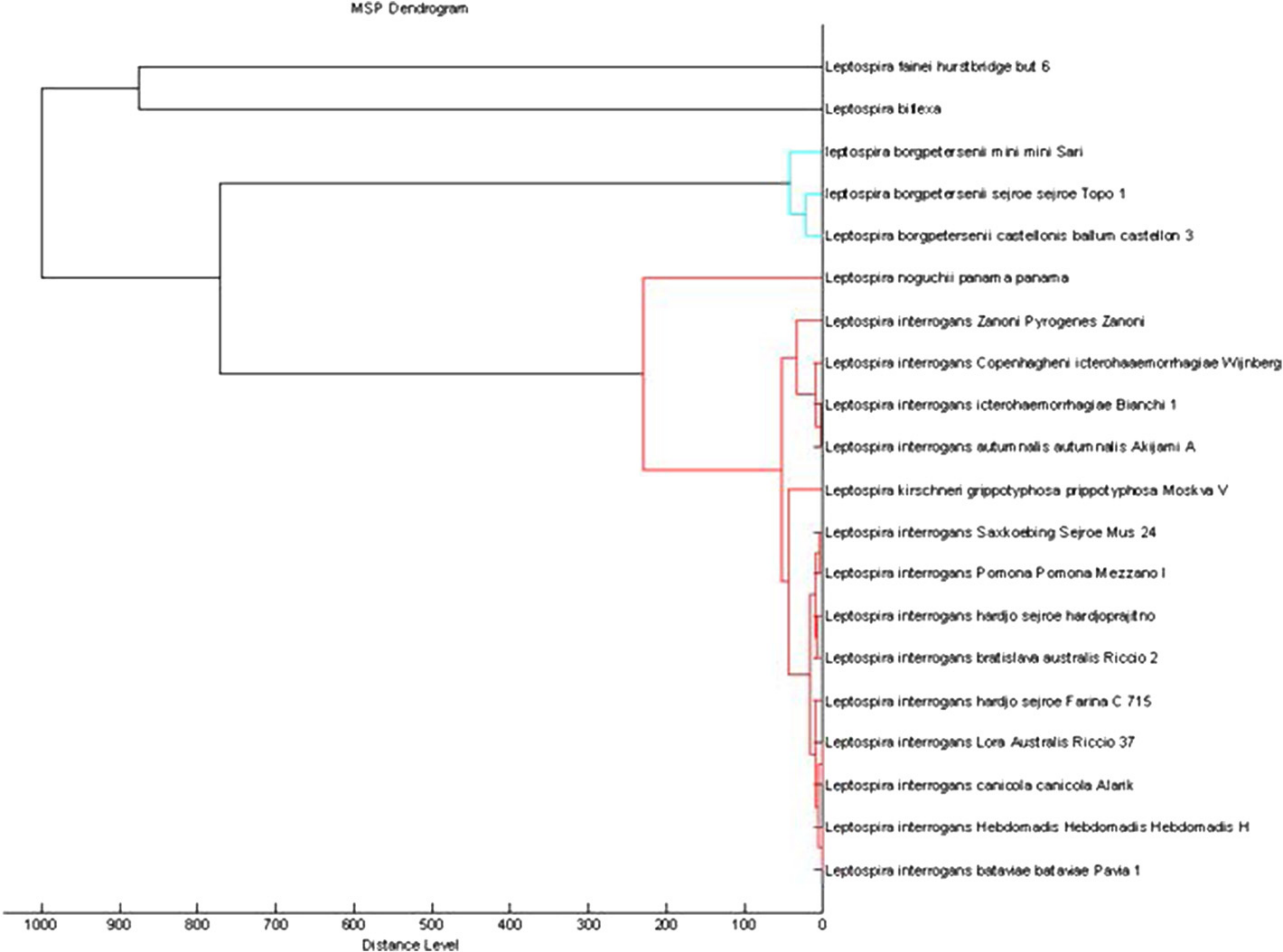
**“Main Spectra Profiles”-based dendrogram of the 20 *Leptospira *** **sp. reference strains analysed in this study.**

Using the software MALDI Biotyper version 3.1 the 20 *Leptospira* protein reference spectra were visualized in a dendrogram (Figure 2). *L. biflexa* and *L. fainei* were located in a branch while *L. interrogans*, *L. kirschneri, L. noguchii* and *L. borgpetersenii* clusterized in a separate one. The latter was further divided into two groups respectively containing, *L. borgpetersenii* on one side and *L. interrogans*, *L. kirschneri,* and *L. noguchii* on the other side.

### ClinProTools statistical analysis

**Figure 3:**
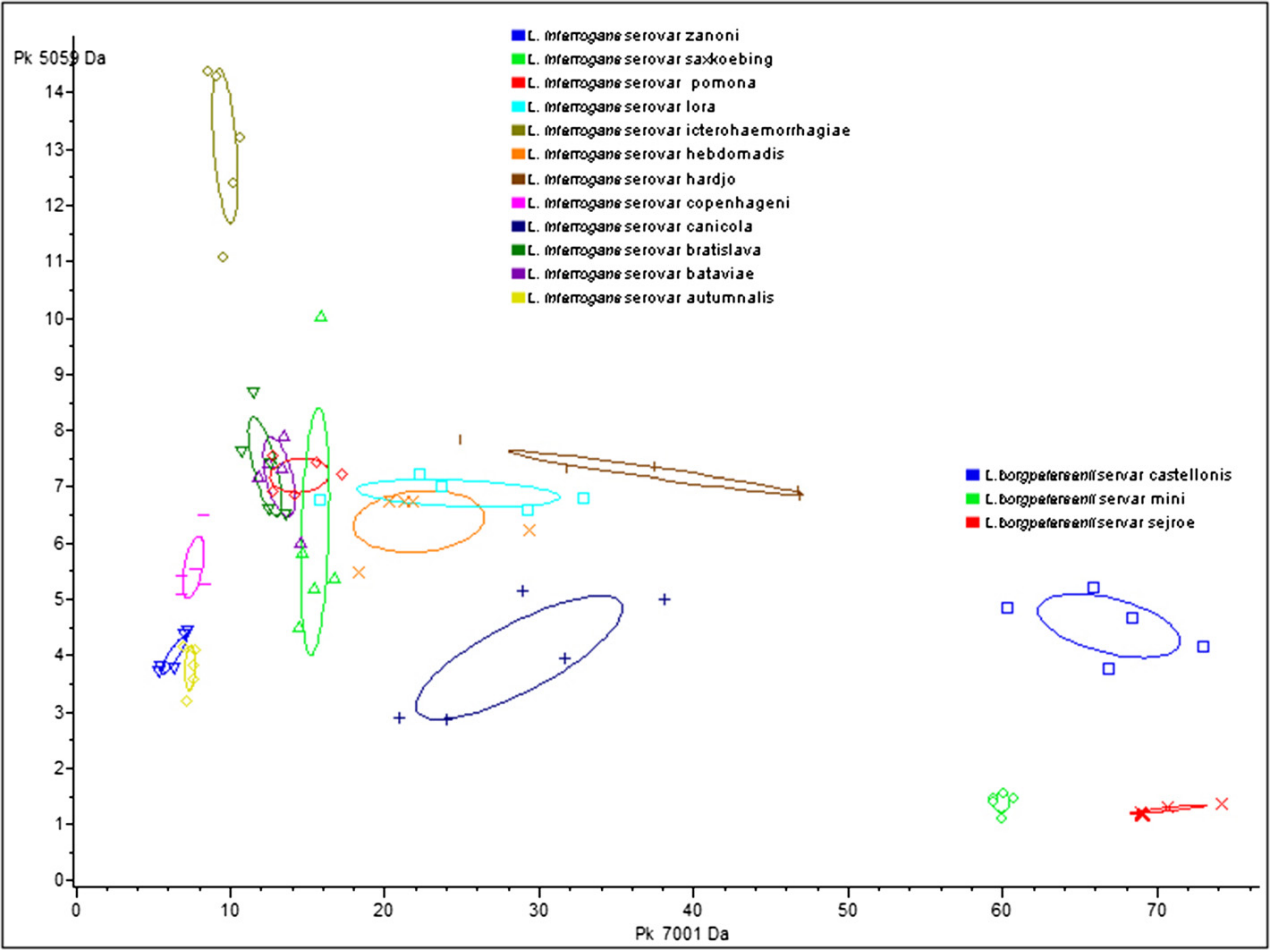
**Differentiation of *L*. *interrogans *and *L*. *borgpetersenii *serovars by ClinProTools software.** “2D Peak Distribution View” displaying the distribution of the different 15 serovars (by way of example, 5 replicates per serovar are shown) analysed on the basis of the first two best discriminating peaks among the spectra of all serovars considered. The symbols represent the ratio area/intensity with respect to the two peaks considered for each spectrum, the same symbols represent replicates of the same serovar and the ellipses represent the standard deviation within the same serovar.

In order to visualize and identify the discriminatory peaks among the different serovars of *L. interrogans* and *L. borgpetersenii* analysed in this study, the spectra used for the MSP spectra creation were analysed by ClinProTools software and each replicates were shown on a two-dimensional plane; by default the first two best discriminating peaks of the current statistic sort order were displayed (Figure 3).

In order to identify specific peaks able to discriminate at serovar level, the spectra obtained for the species *L. interrogans* and *L. borgpetersenii,* respectively, were analysed by the statistical software.

The whole of the spectra of the strains within the same species were analysed applying different algorithms available in the software. For the species *L. interrogans* the SNN model showed a RC of 100% with a CV of 79.29% with 20 discriminating peaks that are reported in Table 2. In particular, the serovar Autumnalis was characterized by the presence of only 3 peaks (3,684 Da, 5,527 Da, and 11,049 Da), the serovar Bratislava by the presence of all the peaks except for the peak of 5,671 Da and 6,915 Da. Analogously, for the remaining serovars the combinations of the presence or the absence of the discriminating peaks shown in Table 2 resulted to be discriminatory among serovars.

**Table 2:** **Differentiating peaks obtained by statistical analysis of 12 serovars belonging to the species *L*. *interrogans***

**Serovars**	**Peak mass (m/z) representing the protein size in Dalton**																			
	**3,506**	**3,684**	**3,952**	**5,059**	**5,527**	**5,658**	**5,671**	**5,782**	**6,193**	**6,284**	**6,356**	**6,915**	**6,941**	**6,970**	**7,013**	**7,059**	**7,077**	**7,902**	**11,049**	**11,311**
Autumnalis	-	+	-	-	+	-	-	-	-	-	-	-	-	-	-	-	-	-	+	-
Canicola	§	-	§	-	-	§	-	§	+	§	-	+	+	+	+	+	+	§	-	§
Icterohaemorrhagiae	§	+	+	+	+	§	-	+	-	-	+	-	-	§	+	§	+	+	+	+
Hardjo	+	-	+	+	-	-	+	+	+	+	+	+	+	+	+	+	+	§	-	§
Hebdomadis	§	+	+	+	-	-	-	§	+	-	-	-	§	§	-	+	+	§	-	-
Pomona	+	+	+	+	-	-	-	+	+	+	+	-	§	+	+	+	+	+	-	-
Saxkoebing	-	-	§	+	-	-	-	§	-	§	§	-	§	+	+	+	+	+	+	§
Batavie	+	+	§	+	+	+	+	-	+	-	-	+	+	+	+	+	+	§	+	+
Bratislava	+	+	+	+	+	+	§	+	+	+	+	§	+	+	+	+	+	+	+	+
Lora	-	-	+	§	-	-	+	+	§	+	+	-	-	-	-	§	§	+	-	-
Copenhageni	-	+	+	§	+	-	-	-	+	+	-	-	-	-	-	-	§	§	+	-
Zanoni	-	+	-	-	+	+	+	-	-	-	-	+	+	-	-	-	-	-	§	+

The identification of each serovar is derived from the combinations of the 20 discriminating considered peaks.

+: peak with area/intensity of the Serovar Average Spectrum higher than the Total Average Spectrum for the respective considered peak.

-: no peak found.

§: peak with area/intensity of the Serovar Average Spectrum lower than the Total Average Spectrum for the respective considered peak.

Discriminating peaks occurred also within the species *L. borgpetersenii* (Table 3). In particular, the presence of the peak of 3,024 Da was characteristic of the serovar Mini, the absence of the peak of 8,068 Da was characteristic of the serovar Sejroe.

**Table 3:** **Differentiating peaks obtained by statistical analysis of 3 serovars belonging to the species *L*. *borgpetersenii***

**Serovars**	**Peak mass (m/z) representing the protein size in Dalton**				
	**3,024**	**3,499**	**6,728**	**7,357**	**8,068**
Castellonis	-	+	§	+	+
Mini	+	§	+	-	+
Sejroe	-	+	+	+	-

The identification of each serovar is derived from the combinations of the 5 discriminating considered peaks.

+: peak with area/intensity of the Serovar Average Spectrum higher than the Total Average Spectrum for the respective considered peak.

-: no peak found.

§: peak with area/intensity of the Serovar Average Spectrum lower than the Total Average Spectrum for the respective considered peak.

## Discussion

There is an urgent need for new diagnostic tools and typing methods easy to perform and readily available for public health and research laboratories for the important but neglected and understudied human disease that is leptospirosis: MALDI-TOF MS applied to the identification of leptospires described in this study enables simplicity, rapidity and cost-effectiveness that are unachievable with current technologies utilized for the typing of these bacteria. MALDI-TOF MS was already successfully applied to the identification of *Leptospira* sp. strains by Djelouadji et al. [7] and Rettinger et al. [24]. In this study, we made a further contribution to evaluate the usefulness of MALDI-TOF MS in the field of leptospirosis and we accomplished two objectives: i) to supplement our Bruker Daltonics database by using our own panel of leptospires including reference pathogenic and saprophytic strains circulating in Italy in order to assess the possible application of MALDI-TOF MS to the diagnosis of leptospirosis in our reference laboratory; ii) to create in our laboratory a statistical model able to differentiate at serovar level, by discriminating peaks, unknown strains belonging to *L. interrogans* and *L. borgpetersenii* species, respectively for epidemiological purposes.

As reported also by Rettinger et al. [24], by using a very simple protocol we obtained an interpretable identifying protein profile for each of the 20 analysed strains starting from a minimal concentration of 1×10^6^ organisms/ml that is achieved with no special requirements of growth conditions: this indicates the possibility of using early *Leptospira* cultures to perform MALDI-TOF MS consequently reducing the delay for their identification. Moreover, we further observed that profiles were robust, being independent from the EMJH lot used for cultivation.

In this study we were able to group by Flex Analysis software the spectra obtained by MALDI-TOF MS of the 20 reference strains analysed into 4 main classes corresponding to the 6 *Leptospira* sp. showing species-specific protein profiles with unique peaks in each species analysed. Moreover, it was also possible to observe little differences among the profiles of the analysed strains within a given species (namely *L. interrogans* and *L. borgpetersenii*, for which more than one strain was analysed), probably due to the presence of differences among the protein patterns of distinct serovars, as subsequently confirmed using ClinProTools analysis that showed serovar-specific peaks. This observation is related to the strains overall analysed in this study taking into account that the ClinProTools software results are specific for the panel of the strains analysed in each experimental session. For this reason confirming the result obtained by Rettinger et al. [24], we cannot definitely conclude that we identified universal serovar-specific peaks, since we used a selected panel of serovars in this study. Nevertheless, a serovar included in the reference panel of the species analysed could be likely assigned without genetic analysis.

The dendrogram obtained by Maldi Biotyper analysis on the basis of the protein patterns of the six species tested reflects the phylogenetic tree based on 16SrRNA sequencing reported in the literature [24] showing *Leptospira* species clustered according to their pathogenicity (pathogenic and saprophytic strains clearly separated into different clusters) and confirming the comparability of the results obtained by mass spectrometry and by molecular typing methods.

*Leptospira* identification has been traditionally accomplished by serological methods [8] which do not always give unambiguous results. Further, the adoption of a genotypic classification complicated the identification of leptospires because of the same serovars may be found in more than one species and some species contain both pathogenic and non pathogenic serovars [5,7], thus the determination of serovar is no longer sufficient to assign an isolate to its correct species. Actually, for public health purposes it has become essential to identify not only the serovar but also the species of isolates in order to accurately track the transmission of *Leptospira* during outbreaks.

MALDI-TOF MS shows, in the laboratory diagnosis of leptospirosis, as previously reported for other bacterial diseases, several advantages over currently available methods of species-level identification [21,22]. First of all, the minimal sample preparation and simple acquisition combined with its potential for high throughput sample automation make the technique a valuable and rapid identification method (it takes only one hour and thirty minutes to obtain the results) [7,25]. Finally, the method is more cost-effective, with an estimated total cost of 0.50 Euro per strain in our experience. All these considerations put this methodology in a central position in microbiology also in the case of such a neglected pathogen as *Leptospira*.

Our results obtained with 20 reference *Leptospira* strains showed that MALDI-TOF MS followed by the analysis with ClinProTools software could be used as a first-line technique for rapid, cheap and reliable identification of *Leptospira* strains at the species level and possibly to discriminate certain serovars that belong to the same genomospecies, as previously proposed by Rettinger et al. [24]. The MALDI-TOF MS spectra available at the moment will serve as a basis to create a database for further identification of unknown strains at serovar level, and our data strongly suggest that such an application could be likely successful.

In recent years, the development of genetic tools and the availability of complete genome sequences of pathogenic *Leptospira* have made it possible to apply state-of-the-art approaches to determine the virulence mechanisms of leptospires [2]; likewise, MALDI-TOF MS could prove in the future a useful tool to evidence the existence of different biotypes showing different protein profiles, possibly related to different pathogenic virulence factors, to be further investigated by more sophisticated approaches in order to gain an insight into the biology of leptospires.

## Conclusions

MALDI-TOF MS emerged over the last years as a useful technology to be applied in the clinical microbiology setting and it has revolutionized the work-flow also in our laboratory, enabling rapid identification of bacteria and yeasts for clinical diagnosis.

In this study, we confirmed that MALDI-TOF MS could be a powerful method for research and diagnostic in the field of leptospirosis with broad applications ranging from the detection and identification of pathogenic leptospires for diagnostic purposes to the typing of pathogenic and non-pathogenic leptospires for epidemiological purposes in order to enrich our knowledge about the epidemiology of the infection in different areas, taking into account that the typing of the circulating leptospiral strains is the first step towards identifying reservoirs and generating control strategies.

## Competing interests

The authors declare the absence of any competing interests.

## Authors’ contribution

Designed the experiments: AC, GP, CG. Performed the experiments: GP, CG, SM, MB, MP, SR. Analysed the data: AC, FDC, MCA, CC, MCM. Contributed reagents, materials, analysis tools: AC. Wrote the manuscript: AC, GP, CG, SM, MB. Conceived and supervised the project: AC, CC. All authors read and approved the final manuscript.
